# 4-Methyl­anilinium *p*-toluene­sulfonate

**DOI:** 10.1107/S1600536810021537

**Published:** 2010-06-26

**Authors:** Rui-jun Xu

**Affiliations:** a Ordered Matter Science Research Center, College of Chemistry and Chemical Engineering, Southeast University, Nanjing 210096, People’s Republic of China

## Abstract

The crystal structure of the title compound, C_7_H_10_N^+^·C_7_H_7_O_3_S^−^, displays strong N—H⋯O and N—H⋯S hydrogen bonding between the ammonium group and the *p*-toluene­sulfonate anion, linking the cations and anions into chains along the *b* axis.

## Related literature

For background to dielectric–ferroelectric materials, see: Hang *et al.* (2009 ▶); Li *et al.* (2008 ▶).
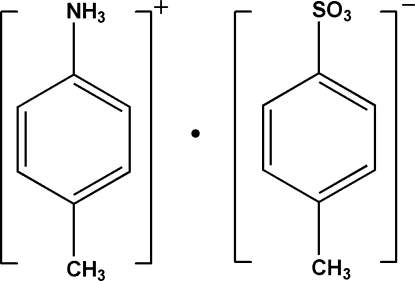


## Experimental

### 

#### Crystal data

C_7_H_10_N^+^·C_7_H_7_O_3_S^−^
*M* *_r_* = 279.35
Monoclinic, 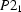

*a* = 5.775 (4) Å
*b* = 9.026 (5) Å
*c* = 13.350 (8) Å
β = 96.344 (9)°
*V* = 691.6 (7) Å^3^
*Z* = 2
Mo *K*α radiation
μ = 0.24 mm^−1^
*T* = 293 K
0.2 × 0.2 × 0.2 mm

#### Data collection

Rigaku Mercury2 diffractometer
Absorption correction: multi-scan (*CrystalClear*; Rigaku, 2005 ▶) *T*_min_ = 0.929, *T*_max_ = 1.000
6641 measured reflections
3136 independent reflections
2876 reflections with *I* > 2σ(*I*)
*R*_int_ = 0.029

#### Refinement

*R*[*F* ^2^ > 2σ(*F* ^2^)] = 0.041
*wR*(*F* ^2^) = 0.093
*S* = 0.99
3136 reflections
174 parameters
1 restraint
H-atom parameters constrained
Δρ_max_ = 0.18 e Å^−3^
Δρ_min_ = −0.23 e Å^−3^
Absolute structure: Flack (1983 ▶), 1448 Friedel pairs
Flack parameter: 0.05 (8)

### 

Data collection: *CrystalClear* (Rigaku, 2005 ▶); cell refinement: *CrystalClear*; data reduction: *CrystalClear*; program(s) used to solve structure: *SHELXS97* (Sheldrick, 2008 ▶); program(s) used to refine structure: *SHELXL97* (Sheldrick, 2008 ▶); molecular graphics: *SHELXTL* (Sheldrick, 2008 ▶); software used to prepare material for publication: *PRPKAPPA* (Ferguson, 1999 ▶).

## Supplementary Material

Crystal structure: contains datablocks I, global. DOI: 10.1107/S1600536810021537/fj2307sup1.cif

Structure factors: contains datablocks I. DOI: 10.1107/S1600536810021537/fj2307Isup2.hkl

Additional supplementary materials:  crystallographic information; 3D view; checkCIF report

## Figures and Tables

**Table 1:** Hydrogen-bond geometry (Å, °)

*D*—H⋯*A*	*D*—H	H⋯*A*	*D*⋯*A*	*D*—H⋯*A*
N1—H1*D*⋯O1^i^	0.89	2.31	3.170 (3)	164
N1—H1*D*⋯O2^i^	0.89	2.33	2.824 (3)	115
N1—H1*D*⋯S1^i^	0.89	2.81	3.570 (3)	144
N1—H1*E*⋯O1^ii^	0.89	1.96	2.829 (3)	165
N1—H1*F*⋯O3^iii^	0.89	2.02	2.785 (3)	143

Symmetry codes: (i) 
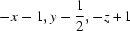
; (ii) 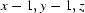; (iii) 
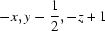
.

**Fig. 1.**
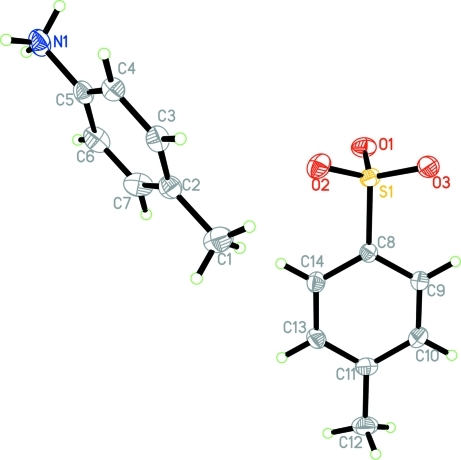
The molecular structure of the title compound, showing the atomic numbering scheme. Displacement ellipsoids are drawn at the 30% probability level.

**Fig. 2.**
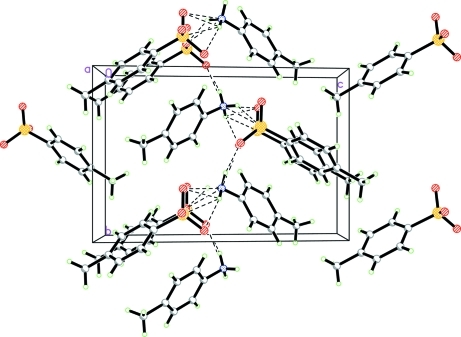
A view of the packing of the title compound, stacking along the b axis. Dashed lines indicate hydrogen bonds.

## supplementary crystallographic information

### Comment

Dielectric-ferroelectric as an interesting class of materials, there are organic ligands (Li *et al.*, 2008), metal-organic coordination compounds (Hang *et al.*, 2009) and organic-inorganic hybrid. In this article, the preparation and crystal structure of the title compound have been presented. It should be not a real ferroelectrics or there may be no distinct phase transition occurred within the measured temperature range. Similarly, below the melting point (477 K) of the compound, the dielectric constant as a function of temperature also goes smoothly, and there is no dielectric anomaly observed.

The asymmetric unit of the title compound contains a (CH_3_—C_6_H_4_—NH_3_^+^) cation and an (CH_3_—C_6_H_4_—SO_3_^-^) anion (Fig.1). The strong N—H···S, N—H···O hydrogen bonds involving H1D and H1E (N1···S1 3.570 (3) Å and N1···O1 2.829 (3) Å) are beneficial to the stability of the crystal structure and link the cations and anions to chains along the *b* axis (Fig. 2 and Tab. 1).

### Experimental

The title compound was obtained by the addition of *p*-toluenesulfonic acid (3.78 g, 0.022 mol) to a solution of 4-methylaniline (2.14 g, 0.02 mol) in ethanol, in the stoichiometric ratio 1.1:1. After two weeks, good quality single crystals were obtained by slow evaporation.

### Refinement

Positional parameters of all the H atoms were calculated geometrically and the H atoms were set to ride on the C and N atoms to which they are bonded, with *U*_iso_(H) = 1.2Ueq(C or N).

### Crystal data

**Table:**

C_7_H_10_N^+^·C_7_H_7_O_3_S^−^	*F*(000) = 296
*M**_r_* = 279.35	*D*_x_ = 1.341 Mg m^−^^3^
Monoclinic, *P*2_1_	Mo *K*α radiation, λ = 0.71073 Å
Hall symbol: P 2yb	Cell parameters from 3136 reflections
*a* = 5.775 (4) Å	θ = 3.6–27.5°
*b* = 9.026 (5) Å	µ = 0.24 mm^−^^1^
*c* = 13.350 (8) Å	*T* = 293 K
β = 96.344 (9)°	Prism, colorless
*V* = 691.6 (7) Å^3^	0.2 × 0.2 × 0.2 mm
*Z* = 2	

### Data collection

**Table:**

Rigaku Mercury2 diffractometer	3136 independent reflections
Radiation source: fine-focus sealed tube	2876 reflections with *I* > 2σ(*I*)
graphite	*R*_int_ = 0.029
Detector resolution: 13.6612 pixels mm^-1^	θ_max_ = 27.5°, θ_min_ = 3.6°
CCD_Profile_fitting scans	*h* = −7→7
Absorption correction: multi-scan (*CrystalClear*; Rigaku, 2005)	*k* = −11→11
*T*_min_ = 0.929, *T*_max_ = 1.000	*l* = −17→17
6641 measured reflections	

### Refinement

**Table:**

Refinement on *F*^2^	Secondary atom site location: difference Fourier map
Least-squares matrix: full	Hydrogen site location: inferred from neighbouring sites
*R*[*F*^2^ > 2σ(*F*^2^)] = 0.041	H-atom parameters constrained
*wR*(*F*^2^) = 0.093	*w* = 1/[σ^2^(*F*_o_^2^) + (0.0447*P*)^2^ + 0.128*P*] where *P* = (*F*_o_^2^ + 2*F*_c_^2^)/3
*S* = 0.99	(Δ/σ)_max_ < 0.001
3136 reflections	Δρ_max_ = 0.18 e Å^−^^3^
174 parameters	Δρ_min_ = −0.23 e Å^−^^3^
1 restraint	Absolute structure: Flack (1983), 1448 Friedel pairs
Primary atom site location: structure-invariant direct methods	Flack parameter: 0.05 (8)

### Special details

**Table:**

Geometry. All e.s.d.'s (except the e.s.d. in the dihedral angle between two l.s. planes) are estimated using the full covariance matrix. The cell e.s.d.'s are taken into account individually in the estimation of e.s.d.'s in distances, angles and torsion angles; correlations between e.s.d.'s in cell parameters are only used when they are defined by crystal symmetry. An approximate (isotropic) treatment of cell e.s.d.'s is used for estimating e.s.d.'s involving l.s. planes.
Refinement. Refinement of *F*^2^ against ALL reflections. The weighted *R*-factor *wR* and goodness of fit *S* are based on *F*^2^, conventional *R*-factors *R* are based on *F*, with *F* set to zero for negative *F*^2^. The threshold expression of *F*^2^ > σ(*F*^2^) is used only for calculating *R*-factors(gt) *etc*. and is not relevant to the choice of reflections for refinement. *R*-factors based on *F*^2^ are statistically about twice as large as those based on *F*, and *R*- factors based on ALL data will be even larger.

### Fractional atomic coordinates and isotropic or equivalent isotropic displacement parameters (Å^2^)

**Table:**

	*x*	*y*	*z*	*U*_iso_*/*U*_eq_	
S1	0.14320 (9)	−0.16236 (6)	0.34803 (4)	0.03362 (14)	
O1	0.0753 (3)	−0.0673 (2)	0.42886 (12)	0.0456 (4)	
O2	0.0234 (3)	−0.30349 (19)	0.34704 (14)	0.0481 (4)	
O3	0.3936 (2)	−0.1756 (2)	0.34883 (12)	0.0485 (4)	
C8	0.0436 (3)	−0.0720 (2)	0.23353 (15)	0.0302 (4)	
C9	0.1872 (4)	0.0278 (3)	0.19156 (17)	0.0363 (5)	
H9A	0.3372	0.0450	0.2223	0.044*	
C10	0.1071 (4)	0.1023 (3)	0.10348 (19)	0.0420 (6)	
H10A	0.2038	0.1701	0.0761	0.050*	
C11	−0.1161 (4)	0.0769 (3)	0.05557 (17)	0.0394 (5)	
C12	−0.2047 (5)	0.1588 (4)	−0.0397 (2)	0.0589 (8)	
H12A	−0.3718	0.1546	−0.0491	0.071*	
H12B	−0.1434	0.1134	−0.0962	0.071*	
H12C	−0.1553	0.2603	−0.0343	0.071*	
C13	−0.2562 (4)	−0.0238 (3)	0.09898 (18)	0.0405 (6)	
H13A	−0.4056	−0.0420	0.0679	0.049*	
C14	−0.1806 (4)	−0.0981 (3)	0.18702 (18)	0.0368 (5)	
H14A	−0.2783	−0.1647	0.2149	0.044*	
N1	−0.7130 (4)	−0.8010 (3)	0.50369 (16)	0.0513 (5)	
H1D	−0.8346	−0.7437	0.5125	0.062*	
H1E	−0.7621	−0.8925	0.4885	0.062*	
H1F	−0.6154	−0.8026	0.5602	0.062*	
C1	−0.2340 (5)	−0.5599 (4)	0.1861 (2)	0.0590 (7)	
H1A	−0.2324	−0.4538	0.1911	0.071*	
H1B	−0.3120	−0.5889	0.1218	0.071*	
H1C	−0.0768	−0.5963	0.1926	0.071*	
C2	−0.3611 (4)	−0.6242 (2)	0.26934 (18)	0.0408 (6)	
C3	−0.2853 (4)	−0.7531 (3)	0.31786 (18)	0.0400 (5)	
H3A	−0.1548	−0.8010	0.2985	0.048*	
C4	−0.3986 (4)	−0.8132 (3)	0.39495 (18)	0.0392 (5)	
H4A	−0.3448	−0.8996	0.4275	0.047*	
C5	−0.5937 (4)	−0.7409 (3)	0.42196 (17)	0.0385 (5)	
C6	−0.6736 (4)	−0.6122 (3)	0.3749 (2)	0.0485 (7)	
H6A	−0.8047	−0.5645	0.3939	0.058*	
C7	−0.5565 (5)	−0.5552 (3)	0.2994 (2)	0.0508 (6)	
H7A	−0.6097	−0.4680	0.2677	0.061*	

### Atomic displacement parameters (Å^2^)

**Table:**

	*U*^11^	*U*^22^	*U*^33^	*U*^12^	*U*^13^	*U*^23^
S1	0.0322 (2)	0.0360 (3)	0.0334 (3)	0.0060 (3)	0.00711 (18)	0.0046 (2)
O1	0.0537 (10)	0.0513 (11)	0.0326 (9)	0.0096 (9)	0.0085 (7)	−0.0025 (8)
O2	0.0532 (11)	0.0334 (9)	0.0589 (11)	0.0011 (8)	0.0121 (8)	0.0090 (8)
O3	0.0311 (7)	0.0656 (11)	0.0489 (9)	0.0115 (9)	0.0046 (6)	0.0178 (9)
C8	0.0276 (10)	0.0320 (11)	0.0315 (10)	0.0028 (9)	0.0058 (8)	−0.0017 (9)
C9	0.0309 (11)	0.0432 (13)	0.0349 (12)	−0.0018 (10)	0.0044 (9)	0.0023 (10)
C10	0.0416 (13)	0.0448 (14)	0.0409 (13)	−0.0050 (11)	0.0101 (10)	0.0068 (11)
C11	0.0438 (12)	0.0443 (13)	0.0303 (11)	0.0072 (11)	0.0042 (10)	0.0023 (10)
C12	0.0641 (18)	0.074 (2)	0.0371 (14)	0.0073 (16)	0.0010 (12)	0.0134 (14)
C13	0.0295 (11)	0.0532 (15)	0.0380 (12)	0.0016 (11)	−0.0004 (9)	−0.0003 (11)
C14	0.0305 (11)	0.0392 (12)	0.0415 (13)	−0.0011 (10)	0.0071 (9)	0.0019 (10)
N1	0.0401 (11)	0.0737 (14)	0.0412 (11)	−0.0133 (11)	0.0088 (9)	−0.0154 (11)
C1	0.0736 (19)	0.0558 (17)	0.0486 (16)	−0.0024 (16)	0.0105 (14)	0.0017 (14)
C2	0.0469 (13)	0.0390 (14)	0.0362 (12)	−0.0028 (10)	0.0027 (10)	−0.0083 (9)
C3	0.0349 (12)	0.0416 (14)	0.0444 (13)	0.0025 (10)	0.0085 (10)	−0.0099 (11)
C4	0.0392 (12)	0.0377 (12)	0.0402 (12)	0.0013 (10)	0.0026 (9)	−0.0019 (10)
C5	0.0310 (11)	0.0513 (14)	0.0333 (11)	−0.0057 (10)	0.0047 (9)	−0.0119 (11)
C6	0.0385 (13)	0.0572 (16)	0.0494 (15)	0.0153 (11)	0.0032 (11)	−0.0145 (12)
C7	0.0583 (16)	0.0456 (15)	0.0474 (14)	0.0186 (13)	0.0012 (12)	−0.0013 (12)

### Geometric parameters (Å, °)

**Table:**

S1—O2	1.449 (2)	N1—C5	1.458 (3)
S1—O3	1.4500 (18)	N1—H1D	0.8903
S1—O1	1.4655 (18)	N1—H1E	0.8893
S1—C8	1.772 (2)	N1—H1F	0.8904
C8—C9	1.384 (3)	C1—C2	1.514 (4)
C8—C14	1.393 (3)	C1—H1A	0.9600
C9—C10	1.389 (3)	C1—H1B	0.9600
C9—H9A	0.9300	C1—H1C	0.9600
C10—C11	1.393 (4)	C2—C3	1.379 (3)
C10—H10A	0.9300	C2—C7	1.387 (4)
C11—C13	1.386 (3)	C3—C4	1.389 (3)
C11—C12	1.510 (3)	C3—H3A	0.9300
C12—H12A	0.9600	C4—C5	1.384 (3)
C12—H12B	0.9600	C4—H4A	0.9300
C12—H12C	0.9600	C5—C6	1.376 (4)
C13—C14	1.381 (3)	C6—C7	1.374 (4)
C13—H13A	0.9300	C6—H6A	0.9300
C14—H14A	0.9300	C7—H7A	0.9300
			
O2—S1—O3	113.73 (12)	C5—N1—H1D	109.1
O2—S1—O1	110.78 (11)	C5—N1—H1E	109.9
O3—S1—O1	113.02 (11)	H1D—N1—H1E	109.5
O2—S1—C8	106.72 (11)	C5—N1—H1F	109.4
O3—S1—C8	105.82 (10)	H1D—N1—H1F	109.4
O1—S1—C8	106.15 (11)	H1E—N1—H1F	109.5
C9—C8—C14	119.9 (2)	C2—C1—H1A	109.5
C9—C8—S1	119.82 (16)	C2—C1—H1B	109.5
C14—C8—S1	120.26 (17)	H1A—C1—H1B	109.5
C8—C9—C10	120.0 (2)	C2—C1—H1C	109.5
C8—C9—H9A	120.0	H1A—C1—H1C	109.5
C10—C9—H9A	120.0	H1B—C1—H1C	109.5
C9—C10—C11	120.9 (2)	C3—C2—C7	118.0 (2)
C9—C10—H10A	119.5	C3—C2—C1	120.9 (2)
C11—C10—H10A	119.5	C7—C2—C1	121.1 (2)
C13—C11—C10	118.0 (2)	C2—C3—C4	121.8 (2)
C13—C11—C12	120.9 (2)	C2—C3—H3A	119.1
C10—C11—C12	121.1 (2)	C4—C3—H3A	119.1
C11—C12—H12A	109.5	C5—C4—C3	118.2 (2)
C11—C12—H12B	109.5	C5—C4—H4A	120.9
H12A—C12—H12B	109.5	C3—C4—H4A	120.9
C11—C12—H12C	109.5	C6—C5—C4	121.4 (2)
H12A—C12—H12C	109.5	C6—C5—N1	119.6 (2)
H12B—C12—H12C	109.5	C4—C5—N1	119.0 (2)
C14—C13—C11	122.0 (2)	C7—C6—C5	118.9 (2)
C14—C13—H13A	119.0	C7—C6—H6A	120.5
C11—C13—H13A	119.0	C5—C6—H6A	120.5
C13—C14—C8	119.2 (2)	C6—C7—C2	121.8 (2)
C13—C14—H14A	120.4	C6—C7—H7A	119.1
C8—C14—H14A	120.4	C2—C7—H7A	119.1
			
O2—S1—C8—C9	−151.80 (18)	C11—C13—C14—C8	−0.4 (4)
O3—S1—C8—C9	−30.3 (2)	C9—C8—C14—C13	0.3 (3)
O1—S1—C8—C9	90.00 (19)	S1—C8—C14—C13	178.57 (18)
O2—S1—C8—C14	29.9 (2)	C7—C2—C3—C4	−0.2 (3)
O3—S1—C8—C14	151.35 (19)	C1—C2—C3—C4	179.5 (2)
O1—S1—C8—C14	−88.3 (2)	C2—C3—C4—C5	0.6 (3)
C14—C8—C9—C10	0.3 (3)	C3—C4—C5—C6	−0.6 (3)
S1—C8—C9—C10	−178.00 (19)	C3—C4—C5—N1	−179.1 (2)
C8—C9—C10—C11	−0.7 (4)	C4—C5—C6—C7	0.2 (4)
C9—C10—C11—C13	0.6 (4)	N1—C5—C6—C7	178.6 (2)
C9—C10—C11—C12	179.4 (2)	C5—C6—C7—C2	0.3 (4)
C10—C11—C13—C14	0.0 (4)	C3—C2—C7—C6	−0.2 (4)
C12—C11—C13—C14	−178.8 (2)	C1—C2—C7—C6	−179.9 (2)

### Hydrogen-bond geometry (Å, °)

**Table:**

*D*—H···*A*	*D*—H	H···*A*	*D*···*A*	*D*—H···*A*
N1—H1D···O1^i^	0.89	2.31	3.170 (3)	164
N1—H1D···O2^i^	0.89	2.33	2.824 (3)	115
N1—H1D···S1^i^	0.89	2.81	3.570 (3)	144
N1—H1E···O1^ii^	0.89	1.96	2.829 (3)	165
N1—H1F···O3^iii^	0.89	2.02	2.785 (3)	143

Symmetry codes: (i) −*x*−1, *y*−1/2, −*z*+1; (ii) *x*−1, *y*−1, *z*; (iii) −*x*, *y*−1/2, −*z*+1.
